# Structural Deformation of Sm@C_88_ under High Pressure

**DOI:** 10.1038/srep13398

**Published:** 2015-08-25

**Authors:** Jinxing Cui, Mingguang Yao, Hua Yang, Ziyang Liu, Fengxian Ma, Quanjun Li, Ran Liu, Bo Zou, Tian Cui, Zhenxian Liu, Bertil Sundqvist, Bingbing Liu

**Affiliations:** 1 State Key Laboratory of Superhard Materials, Jilin University, No. 2699 Qianjin Street, Changchun 130012, P.R. China; 2 College of Materials Science and Engineering, China Jiliang University, No. 258 Xueyuan Street, Hangzhou 310018, P.R. China; 3 Geophysical Laboratory, Carnegie Institution of Washington, 5251 Broad Branch Road, NW, Washington, DC 20015, USA; 4 Department of Physics, Umeå University, 901 87 Umeå, Sweden

## Abstract

We have studied the structural transformation of Sm@C_88_ under pressure up to 18 GPa by infrared spectroscopy combined with theoretical simulations. The infrared-active vibrational modes of Sm@C_88_ at ambient conditions have been assigned for the first time. Pressure-induced blue and red shifts of the corresponding vibrational modes indicate an anisotropic deformation of the carbon cage upon compression. We propose that the carbon cage changes from ellipsoidal to approximately spherical around 7 GPa. A smaller deformation of the carbon bonds in the area close to the Sm atom in the cage suggests that the trapped Sm atom plays a role in minimizing the compression of the adjacent bonds. Pressure induced a significant reduction of the band gap of the crystal. The HOMO-LUMO gap of the Sm@C_88_ molecule decreases remarkably at 7 GPa as the carbon cage is deformed. Also, compression enhances intermolecular interactions and causes a widening of the energy bands. Both effects decrease the band gap of the sample. The carbon cage deforms significantly above 7 GPa, from spherical to a peanut-like shape and collapses at 18 GPa.

Endohedral metallofullerenes (EMFs) have been attracting much attention because of their potential applications in the fields of medicine, materials science, physics and novel energy^1^. In EMFs, the metal atoms are trapped in fullerene cages to which they also transfer charge. The properties of EMFs depend strongly on the metal atoms trapped inside the cages^2^. The metal-cage bonding interaction in EMFs is different from the traditional metal ligand bond^3^,^4^. The metal atom is situated near the inner surface of the cage and interacts with its near hemisphere, leaving the other half almost unaffected. The coordination of the metal atom is thus uneven in space, which results in an anisotropic charge distribution around the atom, causing a series of special electronic transfer behaviors^5^. Heath *et al.* reported the formation of the first EMF, La@C_60_ 6, which is unfortunately sensitive to air^7^. The first EMF to be isolated is La@C_82_ which has a larger carbon cage^7^. Subsequently, numerous monometallic endohedral fullerenes containing lanthanide ions have been synthesized and isolated. However, the rather low production efficiency and the isomer mixtures of the high EMFs has hindered studies of the intrinsic structures and properties of EMFs. Recently, Yang *et al.* have developed an efficient method for the synthesis and isolation of high purity single isomers of various high EMFs containing a single samarium ion^8^,^9^,^10^. Very recently, we have isolated Sm@C_88_ from carbon soot obtained by electric arc vaporization of graphite rods containing Sm_2_O_3_. As yet, the fundamental structure and physical properties of Sm@C_88_ have not been investigated. This material offers an ideal model to study the internal structure and understand the metal-cage interaction.

Fullerenes have various potential applications, including use as photoconductive materials^11^,^12^, structural reinforcement materials^13^, and so on. In fact, fullerenes may often deform in the applications, and controllable deformations could result in novel structures and properties. High pressure is an effective way to modify the structure and thus tune the properties of materials^14^. The structural stability and evolution of fullerenes under high pressure has been widely studied^15^,^16^,^17^,^18^. For example, the C_60_ molecule deforms and forms links to nearest neighbors by [2 + 2] cycloaddition to form a 3D polymer at 15 GPa and 600 °C^18^. The hardness of this phase is comparable to that of cubic boron nitride (c-BN). In hydrogenated fullerene (C_60_H_18_), the hydrogen atoms can protect the fullerene cage and the structure of the material from distortion up to 32 GPa, much higher than for the pure C_60_ 19. When solvated fullerene (C_60_*m-xylene) is compressed up to 60 GPa, the C_60_ cages collapse while the material still preserves a long-range ordered structure, containing amorphous carbon clusters as its basic units. This material is ultra-incompressible and hard enough to indent diamond^20^,^21^. These interesting phenomena not only contribute to our understanding on the crystalline structure of solids but also emphasize the importance of exploring the deformation of fullerene cages for better understanding the novel phase transitions. In particular, the deformation process of the fullerene cage and the real molecular morphology after the deformation are very important for understanding the resulting properties of the phases formed. Compared with C_60_, high EMFs have a larger frame and lower symmetry, which may lead to a more obvious deformation of the carbon cage under high pressure and thus provide a good model to study the deformation of carbon cages. However, how EMFs deform under high pressure is still unknown and no information exists on how the confined metal atom affects the structural deformation of the carbon cage. High pressure may tune the metal-cage interaction and make it possible to study the effects of the metal atom on the molecular deformation to improve our understanding of the unique metal-cage interaction. This may bring a new insight into the structural stability and the deformation process of fullerenes at the atomic level, making the prediction of new carbon phases possible.

In this work, the infrared (IR) vibrational modes of Sm@C_88_ have been assigned with the help of theoretical simulations. The cage deformation, structural stabili*t*y and the band gap change of Sm@C_88_ under high pressure have been further studied by IR spectroscopy. Classical molecular dynamics simulations on the Sm@C_88_ crystal and density functional theory (DFT) calculations on the molecule have been performed to understand the transformation of Sm@C_88_ upon compression. We present the structural evolution of Sm@C_88_ and the change of the band gap of the material under high pressure. It has also been found that the trapped metal atom supports the carbon cage against collapse under high pressure.

## Results

### IR spectrum of Sm@C_88_

**Figure 1:**
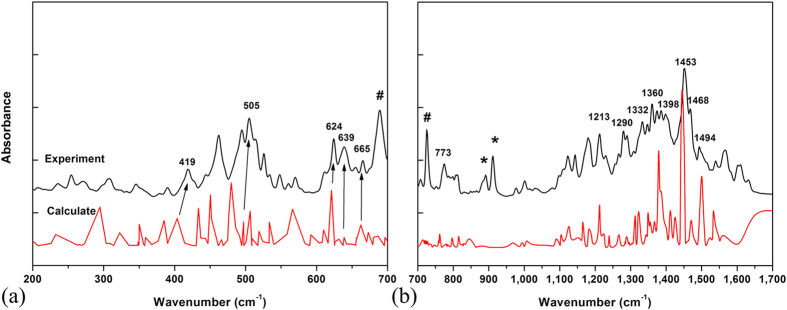
Experimental (black) and calculated (red) infrared spectra of Sm@C_88_ at ambient pressure. (**a**) FIR spectra; (**b**) MIR spectra. The peaks from toluene are marked with symbol “#”. The peaks from ET are marked with asterisks.

**Figure 2:**
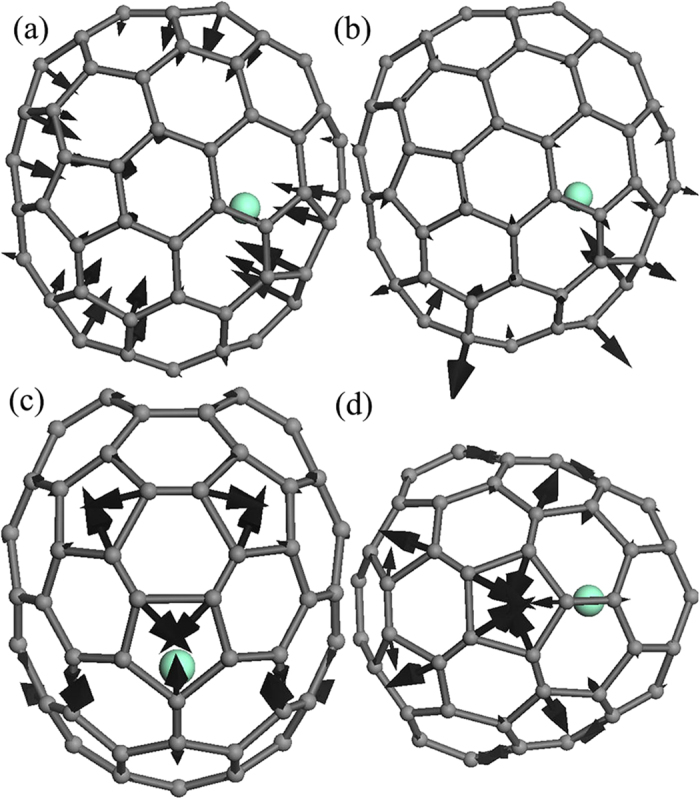
The eigenvectors of IR-active vibrational modes for the bands. (**a**) 419, (**b**) 773, (**c**) 1453 and (**d**) 1468 cm^−1^.

The IR spectrum of the Sm@C_88_ crystals recorded at room temperature and atmospheric pressure is displayed in Fig. 1. No reports exist giving measured IR spectra or the assignment of the IR-active peaks of Sm@C_88_. To understand the origin of the vibrational modes, we have calculated the IR spectrum for Sm@C_88_ with DMOL^3^. Based on this simulation, the IR spectrum can be roughly divided into three regions. (1) The bands from 200 to 450 cm^−1^ are attributed to the radial breathing vibrations of the carbon cage (for example, Fig. 2(a) shows the eigenvector of the vibrational mode at 419 cm^−1^). (2) The bands between 450 and 810 cm^−1^ are from C-C/C=C radial bending vibrations. (3) The bands from 1000 to 1600 cm^−1^ are related to C-C/C=C tangential stretching vibrations on the spherical surface. For example, the mode at 1468 cm^−1^ arises from a stretching vibration of a five-membered ring, while the mode at 1453 cm^−1^ is correlated with the stretching vibration of a six-membered ring. The eigenvectors corresponding to these vibrational modes are shown in Fig. 2(c,d). Both spectra match well with each other in the number and the shape of the peaks except for slight differences in the frequencies in the far-infrared (FIR) range and the IR signals of toluene (marked with symbol “#”) and bis(ethylenedithio)tetrthiafulvalene (ET) (marked with asterisks). This may be due to the interaction between Sm@C_88_ and adjacent molecules. The above assignments of the IR-active vibrational modes are similar to the IR spectral analysis by Yang *et al.* for other endohedral fullerenes^22^,^23^.

**Figure 3:**
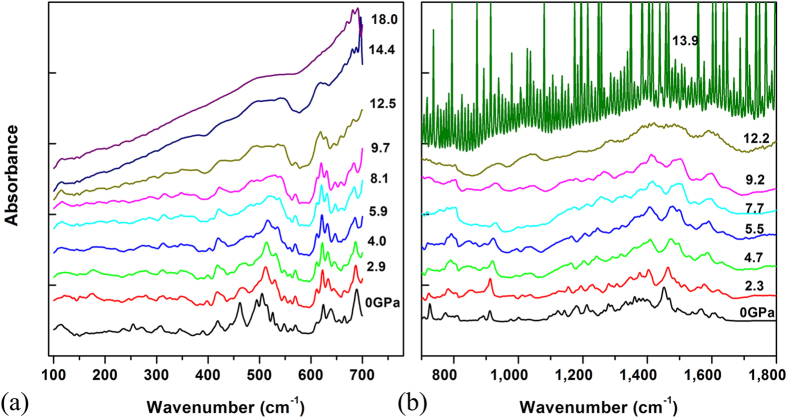
Absorbance spectra of Sm@C_88_ under high pressure. (**a**) FIR region, (**b**) MIR to 1800 cm^−1^. The IR absorbance spectra under high pressure were shifted for clarity.

Figure 3 shows IR spectra of the sample under high pressure up to 18 GPa. Most vibrational modes exhibit blue shifts except a few vibrational modes exhibit a red shift with increasing pressure. The blue shift of the vibrational modes is a common phenomenon in high-pressure studies and results from pressure-induced shrinkage of the bond length. The red shift of the vibrational modes may result from an extension of the corresponding bond lengths and changes of corresponding bond angles with increasing pressure. A pressure-induced red shift of some radial bending vibrations in C_70_ solvates has been observed in previous studies^24^.

With increasing pressure, the intensities of most modes decrease and the peaks broaden significantly. At 18 GPa all modes in the FIR region disappear and only a single broad band is detectable around 500 cm^−1^. This indicates that the carbon cages start to lose the cage feature and collapse. Similar transitions have also been observed in other fullerenes. For example, Wasa *et al.* observed amorphization of C_70_ above 12 GPa^25^. We further used high resolution TEM (HRTEM) to study the detailed structure of the released sample. The HRTEM images of the samples at ambient pressure and decompressed from 18 GPa are shown in Fig. S2. The figures show that the sample at ambient pressure is an order phase, and the decompressed sample is amorphized. This indicates that the sample transforms to amorphization at 18 GPa and can be quenched to ambient pressure, in agreement with our IR results.

**Figure 4:**
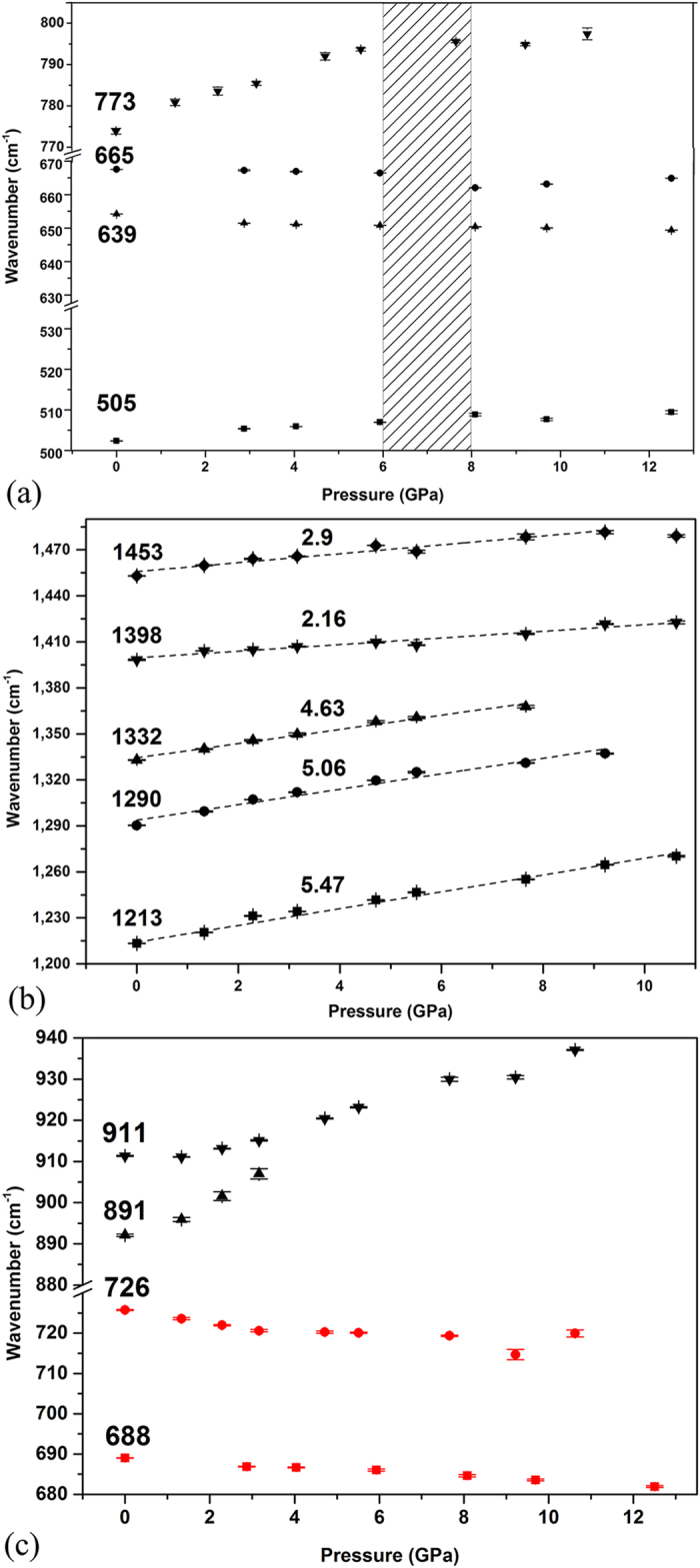
Frequencies of representative IR modes. (**a**) C-C/C=C radial bending vibrations and (**b**) C-C/C=C tangential stretching vibrations of the carbon cage as functions of pressure. The pressure coefficients are shown near the fitted lines. (**c**) The IR modes from ET (black) and toluene (red) as functions of pressure.

It has been suggested that the pressure coefficient (dω/dp) reflects the pressure response of the corresponding vibrational modes, and changes in the pressure coefficients could reflect changes in molecular structure^19^. We can thus analyze the pressure coefficients of the vibrational modes in different parts on the carbon cage to study the deformation of Sm@C_88_ under high pressure. We used Lorentzian functions to fit the IR-active vibrational bands up to 12.5 GPa. The frequencies of four intense C-C/C=C radial bending vibrational bands at 505, 639, 665 and 773 cm^−1^ are plotted as a function of pressure in Fig. 4(a). The modes at 505 and 773 cm^−1^, which are related to the C-C/C=C radial bending vibrations of the top and bottom parts of the cage (the eigenvector of the vibrational mode at 773 cm^−1^ is shown in Fig. 2(b)), shift to higher frequencies. However, the modes at 639 and 665 cm^−1^, which are attributed to the C-C/C=C radial bending vibrations in the horizontal direction of the left and right parts of the cage (the carbon cage is seen from the view of Fig. 2(b)), shift to lower frequencies. Furthermore, the magnitudes of the pressure coefficients of the red-shift modes are smaller than those of blue-shift modes. The pressure coefficients of the vibrational modes start to change in the range 6–8 GPa. We also plotted the pressure dependences of five intense C-C/C=C tangential stretching vibrational modes at 1290, 1332, 1213, 1453 and 1398 cm^−1^ in Fig. 4(b). Comparing with the pressure coefficients of these vibrational modes, we find that the closer the relevant parts of the carbon cage are to the Sm atom, the smaller are the pressure coefficients of the corresponding C-C/C=C tangential vibrational modes. For example, the vibrational mode at 1290 cm^−1^, which is from the part far away from the Sm atom, exhibits a larger pressure coefficient of 5.06 ± 0.31 cm^−1^ GPa^−1^, while the vibrational mode at 1398 cm^−1^, which is from the part close to the Sm atom, exhibits a much smaller pressure coefficient of 2.16 ± 0.16 cm^−1^ GPa^−1^. We also plotted the modes from toluene and ET molecules as a function of pressure in Fig. 4(c). The modes at 688 and 726 cm^−1^ from toluene exhibit a slight red shift, which may result from pressure-induced enhancement of intermolecular van der Waals force between the carbon cage and toluene^19^. The red shift could also result from an extension of the corresponding bond lengths and changes of corresponding bond angles with increasing pressure. The modes at 891 and 911 cm^−1^ from ET exhibit a blue shift, which may be due to compression rather than charge-transfer interaction, because the pressure-induced charge transfer usually results in a red shift in the vibrational modes. Taking into account of the interaction between Sm@C_88_, toluene and ET molecules, the change of the modes of Sm@C_88_ under high pressure may also result from an evolution of the interaction with the molecule environment. To determine the effect of the pressure medium on the transformations of our sample, we performed high-pressure mid-infrared (MIR) experiments with argon and KBr as pressure media, respectively. Both experiments gave very similar results on the pressure evolutions and coefficients of the corresponding vibration modes, which suggests that the observed structural deformations of the carbon cage are due to the effect of compression.

**Figure 5:**
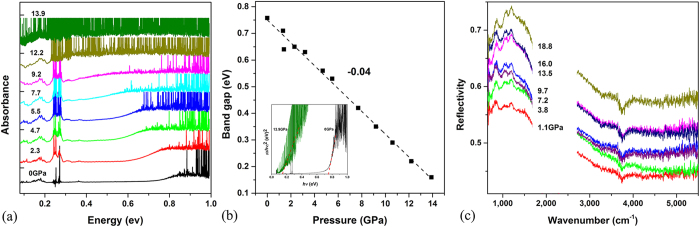
(**a**) The mid-IR region shows the absorption edge under high pressure. (**b**) The band gap as a function of pressure. Inset is the plots of (αhν)^2^ versus hν at ambient pressure and 13.9 GPa. (**c**) IR reflectivity spectra of the sample under high pressure.

High-pressure absorption spectra shown in Fig. 5(a) display clear onset of a broad hump which corresponds to the absorption edge of the sample. This absorption edge gradually shifts to lower energy with increasing pressure and completely moves into FIR region at 13.9 GPa. The indirect band gap E_g_ of the sample can be estimated from the x-axis intercept by extrapolating the linear portion of the (αhν)^2^ versus hν plot to α = 0 (inset of Fig. 5(b)), where α and hν are the absorption coefficient and the incident photon energy. We plotted the band gap as a function of pressure in Fig. 5(b). The band gap decreases almost linearly from 0.73 eV at ambient pressure to 0.16 eV at 13.9 GPa with a pressure coefficient of −0.043 ± 0.001 eVGPa^−1^. It is worth mentioning that the band gaps of solid C_70_^26^ and C_60_^27^ decrease as pressure increases, to a value of 1.4 eV before the molecular collapse, but never reach such low value as observed in our Sm@C_88_. To determine whether the material experiences a metallization transformation, we measured the high-pressure infrared reflectivity spectra of the material as shown in Fig. 5(c). The reflectivity of the sample gradually increased with pressure, which is consistent with the result of the absorption spectra of the sample. However, neither an abrupt increase in reflectivity nor a Drude-Lorentz oscillator model feature in the reflectivity spectra was observed. That suggests that the band gap of the material only decreases to a small value, rather than closes.

### Molecular Deformation of Sm@C_88_

**Figure 6:**
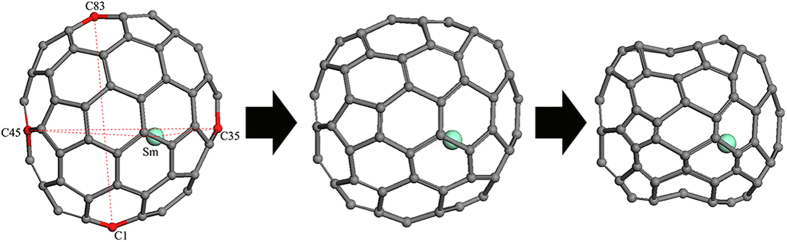
The deformation process of the Sm@C_88_ under high pressure. The major Sm (green) site along with the symmetry plane. C1, C35, C45 and C35 are marked as red.

**Figure 7:**
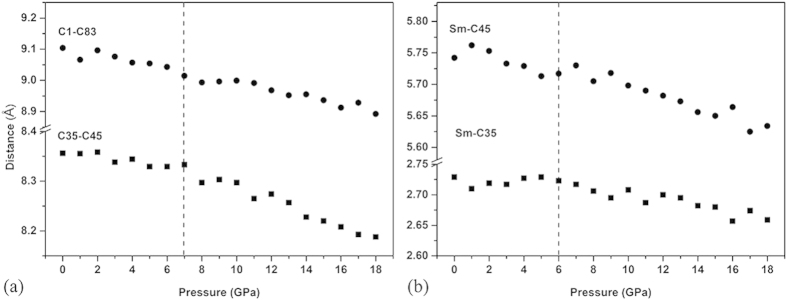
(**a**) Calculated pressure dependences of the C35-C45 and C1-C83 diameters of the carbon cage. (**b**) Calculated pressure dependences of the Sm-C35 and Sm-C45 distances.

In order to explain the deformation process of our Sm@C_88_ under pressure, we performed classical molecular dynamics simulations on Sm@C_88_ crystal. Classical molecular dynamics simulations have been successfully used in studying the deformation of carbon nanotubes under pressure^28^,^29^. The results from the simulations agree well with the experiments. A sketch map of the Sm@C_88_ cage with carbon atoms numbered is shown in Fig. 6. Several characteristic distances that can be used to show the deformation of the carbon cage have been taken into account. The horizontal diameters (distance between C35-C45), the vertical diameters (distance between C1-C83) of the carbon cages and the distances of Sm-C45 (close to Sm) and Sm-C35 (far away from Sm) under high pressure were obtained from the simulations. We found that the pressure derivatives (dR/dp) of these distances start to change at around 7 GPa, as shown in Fig. 7(a). The horizontal diameter (C35-C45) of the carbon cage decreases slowly below 7 GPa, and then decreases at a faster rate above 7 GPa. The vertical diameter (C1-C83) of the carbon cage always shows a larger decrease than the horizontal one. Above 7 GPa, both distances of the carbon cage decrease at a faster rate with increasing pressure than before, demonstrating a more obvious deformation of the carbon cage. Based on the pressure evolutions of these diameters, we propose the following deformation process for the carbon cage. First, the deformation happens in the vertical direction of the cage. Then, the carbon cage changes from an ellipsoidal to an approximately spherical shape, further to a peanut-like shape at higher pressure, and finally collapses. The corresponding deformation process of Sm@C_88_ is shown in Fig. 6. It should point out that our simulations describe a less obvious deformation on the carbon cage than our experimental observations. This might be due to the fact that the classical potential is soft and the simulations usually underestimate the structural changes, as mentioned in the simulations of carbon nanotubes^30^. However, the classical method can give a qualitative description for the structural changes under pressure. Indeed, our simulations give results compatible with our experimental observations. The simulation pressure at which the pressure derivatives of the carbon cage change is consistent with that for an anomalous change in slope in the IR-active vibrational modes observed in our experiment. The anisotropic deformation of the carbon cage given by our simulation can explain the red and blue shifts of the experimental vibrational modes.

From the pressure dependence of the simulated Sm-C distances, shown in Fig. 7(b), we observed that the Sm-C35 (close to Sm) distance decreases at a slower rate than the Sm-C45 (far away from Sm) distance, indicating that the area of the carbon cage closer to the Sm atom deforms less. This can explain the smaller pressure coefficients of the vibrational modes in the area close to the Sm atom. The unbalanced charge distribution on the carbon cage results in a stronger coulomb interaction between the Sm atom and the neighboring C atoms. Such ‘support’ from Sm to the near area of the carbon cage may restrain the change of the corresponding C-C/C=C vibrations with increasing pressure.

### HOMO-LUMO gap of Sm@C_88_

Let us now turn to understand the band gap reduction in our molecular crystals. In an EMF crystal, EMF molecules occupy the lattice sites. The electrons of the samarium atom are localized inside the cage and the electrons of the carbon cage are localized surrounding the carbon cage. The band structure of such a crystal can be estimated by a simple tight-binding approximation model, for which the EMF molecules are considered as a single cell unit. Taking the single atomic s-level as an example, the tight-binding approximation formula is


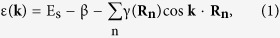


where 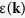
is the Bloch electronic energy. 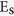 is the single atomic s-level electronic energy, which is the main component of 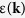, and 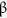 is very small. The width of the energy band depends on 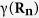, which in turn depends on the interaction and the distance between the neighboring atoms^31^. A stronger interaction produces a wider energy band. Subsequently, in EMF crystals, the single EMF molecular levels broaden into a band due to the interaction between the neighboring molecules. The gap between the highest occupied molecular orbital (HOMO) and the lowest unoccupied molecular orbital (LUMO) determines the band gap between valence band and conduction band of the crystal formed. High pressure can deform the molecular structure and thus decrease the HOMO-LUMO gap of a molecule, to reduce the valence/conduction band gap of the formed crystal, which has been proved in the bisthiaselenazolyl radical dimer^32^. Therefore, the changes of the band gap of the Sm@C_88_ crystal should be related to the observed deformation of the Sm@C_88_ molecule.

**Figure 8:**
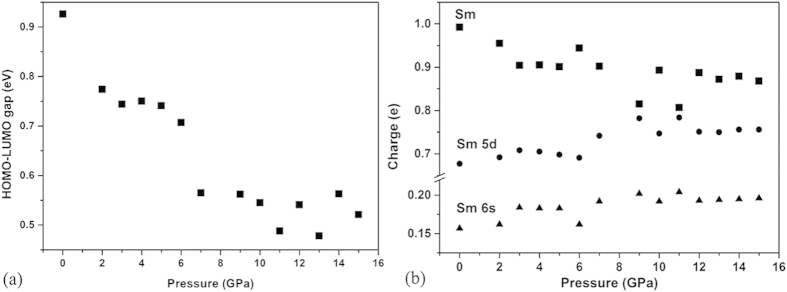
(**a**) Calculated HOMO-LUMO gap as a function of pressure. (**b**) Calculated samarium atomic charge and the electrons on 6s and 5d orbitals of a samarium atom as a function of pressure.

To simulate the corresponding transformation, calculations using the DFT method by DMOL^3^ were conducted on Sm@C_88_ molecules at different pressures. The relative eigenvalues and the gap between HOMO and LUMO of Sm@C_88_ at different pressures have been calculated. The plotted curve for the HOMO-LUMO gap as a function of pressure is shown in Fig. 8(a). It is clear that the HOMO-LUMO gap shows a significant drop (around 0.2 eV) in magnitude at around 7 GPa. Furthermore, we point out that the HOMO of EMF is mostly localized on the cage, while the LUMO is centered on the endohedral metal atom. This has also been demonstrated in other EMF systems, such as Sm_3_@I_h_-C_80_^33^. In our simulations, a significant deformation of the carbon cage and a decrease of the distance between the Sm atom and the carbon cage are observed at around 7 GPa (Fig. 7). Such changes can thus be reasonably related to the reduction of the HOMO-LUMO gap of Sm@C_88_ under pressure. Therefore, the deformation of the carbon cage and the decrease of the Sm-cage distance results in a reduction of the HOMO-LUMO gap of the Sm@C_88_ molecule.

Above 7 GPa, the HOMO-LUMO gap of the Sm@C_88_ molecule is varies slightly with increasing pressure. The reduction of the band gap could arise from the pressure-induced broadening of the valence and conduction bands of the crystal. This can be understood by the fact that the application of pressure compresses the crystal lattice and pushes Sm@C_88_ molecules closer together, thus enhancing the interaction between neighboring molecules. A pressure-induced broadening of the energy bands takes place.

### Charge transfer of Sm@C_88_

In addition to the cage deformation under high pressure, the degree of electron transfer between the entrapped metal and the carbon cage can also contribute to the change of the electronic properties^10^ of Sm@C_88_. Thus we analyzed the Mulliken population of the Sm@C_88_ molecule at different pressures by using *first-principles* with the local density approximation (LDA), shown in Fig. 8(b). The charge on the samarium atom decreases slowly with increasing pressure, suggesting that electrons transfer from the carbon cage to the samarium atom under pressure and that the valence of the carbon cage decreases. We also performed the simulations with the generalized gradient approximation (GGA). The results from the two simulations are similar to each other. To understand the change of the charge transfer, we analyze the composition of orbitals. The pressure dependences of the charge in the 6s and 5d orbitals are depicted in Fig. 8(b). At ambient condition, two 6s electrons of the samarium atom are transferred to the carbon cage, while some electrons from the occupied π orbitals of the cage are fed back to the 5d orbitals of samarium^34^. From Fig. 8(b), we can see that the electron density on 6s and 5d orbitals increases under high pressure. Thus each samarium atom donates fewer electrons, but receives more back-donation from the π orbitals of the carbon cage with increasing pressure. The change of the charge transfer between the Sm atom and the carbon cage may modify the electronic structure and the electron distribution in the material. In another similar system, the alkali-metal-doped fullerides (A_x_C_60_), the amount of charge transferred from alkali metal to carbon cage plays an important role for the electronic properties of the A_x_C_60_ formed. A_4_C_60_ is a small band-gap semiconductor^35^, while A_3_C_60_ is metallic and even superconducting at low temperature^36^. It is also proposed that the metallicity of A_x_C_60_ can be destroyed by a change in the valence of C_60_ and a reduction of the crystal symmetry^37^. The most obvious difference between EMFs and C_60_/C_70_ fullerenes is that metal atoms are trapped inside the former. Thus the change of the charge transfer between the Sm atom and the carbon cage and the decrease of the Sm-cage distance are proposed to be important factors for the reduction of the band gap of Sm@C_88_.

## Conclusions

We have studied the structural stability and deformation of Sm@C_88_ under pressure up to 18 GPa by IR spectroscopy combined with theoretical simulations. We have assigned the experimentally measured IR vibrational modes of Sm@C_88_ at ambient conditions. The carbon cage exhibits anisotropic deformations upon compression and the shape of the carbon cage is proposed to change from ellipsoidal to approximately spherical. The presence of Sm inside the cage plays a role to support the carbon cage against the deformation. Moreover, we observed a significant reduction of the band gap of the material under high pressure. This should be related to the deformation of the carbon cage and the decrease of the Sm-cage distance, which cause a reduction of the HOMO-LUMO gap of the Sm@C_88_ molecule. The enhancement of the intermolecular interaction widens the energy bands, which gives additional contribution to the decrease of the band gap. In addition, the change of the charge transfer from the Sm atom to the carbon cage upon compression modifies the electronic structure and the electron distribution in the material. The carbon cage deforms significantly above 7 GPa, from a spherical to a peanut-like shape. Finally, the carbon cages collapses at 18 GPa.

Taking the EMF Sm@C_88_ as an example, we thus demonstrate the effect of the interaction between the trapped metal atom and the carbon cage on the structural evolution of the hosting carbon cage and the electrical properties under high pressure at the atomic level. This provides a very useful reference for future research on the structures and properties of EMFs and the possibility to create novel, EMF-based functional materials.

## Methods

The angle-dispersive synchrotron X-ray diffraction experiment at ambient condition was performed at 16BMD and 16IDB beamlines at the Advanced Photon Source (APS) of Argonne National Laboratory (ANL). The wavelength of the X-ray was 0.3065 Å. The scanning range was up to 2θ_max_ = 38°. Two-dimensional patterns were radially integrated using the software FIT2D^38^. The X-ray crystallographic study of Sm@C_88_ is shown in the Supplementary Information Part 1. The MIR and FIR high-pressure measurements were carried out at U2A beamline, National Synchrotron Light Source (NSLS), Brookhaven National Laboratory (BNL). The MIR spectra were collected in both transmission and reflection mode by a Bruker Vertex 80v FTIR spectrometer and a Hyperion 2000 IR microscope equipped with a nitrogen-cooled MCT detector. The MIR high-pressure measurements have been repeated with the same type of equipment at State Key Laboratory of Superhard Materials, Jilin University. The FIR absorption spectra were measured using a Bruker IFS 66v FTIR spectrometer and a custom IR microscope equipped with a liquid helium cooled bolometer. Spectral resolution of 4 cm^−1^ and 512 scans applied to all spectra. A symmetrical diamond anvil cell was used with liquid argon as the pressure medium. Samples were loaded into a 100 μm diameter hole drilled in the T301 stainless steel gasket. The pressure was measured with the ruby fluorescence technique^39^. HRTEM images were measured using electron microscopy (JEM-2200FS).

In this work, all *first-principles* calculations (including geometry optimizations, vibrational frequencies, and properties) were carried out by the DMOL^3^ method within the local density approximation (LDA). The classical molecular dynamics (MD) simulations were performed using the Forcite code in Materials Studio using the universal force field (UFF)^40^,^41^. The MD was performed in a NPT ensemble (constant pressure and temperature dynamics) during 0.2 ps (time step = 0.01 fs) in the pressure range from 0 to 18 GPa at room temperature.

## Additional Information

**How to cite this article**: Cui, J. *et al.* Structural Deformation of Sm@C_88_ under High Pressure. *Sci. Rep.* **5**, 13398; doi: 10.1038/srep13398 (2015).

## Supplementary Material

Supplementary Information
