# Supplementary material for: Structural Deformation of Sm@C88 under High Pressure
Source: Sci Rep. 2015 Aug 25;5:13398. doi: 10.1038/srep13398 (PMC4548219; doi:10.1038/srep13398)
Supplement: Supplementary Information [file srep13398-s1.pdf]

Supplementary Information

Structural Deformation of Sm@C_{88} under High Pressure

Jinxing Cui[1], Mingguang Yao[1], Hua Yang[2], Ziyang Liu[2], Fengxian Ma[1], Quanjun Li[1], Ran Liu[1], Bo Zou[1], Tian Cui[1], Zhenxian Liu[3], BertilSundqvist[1, 4], Bingbing Liu[1,*]

[1]State Key Laboratory of Superhard Materials, Jilin University, No. 2699 Qianjin Street, Changchun 130012, P.R. China

[2]College of Materials Science and Engineering, China Jiliang University, No. 258 Xueyuan Street, Hangzhou 310018, P.R. China

[3]Geophysical Laboratory, Carnegie Institution of Washington, 5251 Broad Branch Road, NW, Washington, DC 20015, USA

[4]Department of Physics, Umeå University, 901 87 Umeå, Sweden

Correspondence and requests for materials should be addressed to B.B.L.(liubb@jlu.edu.cn)

Part 1. X-ray crystallographic study of Sm@C_{88}

Part 2. HRTEM study of Sm@C_{88}

Part 3. References for Supplementary Information

Part 1. X-ray crystallographic study of Sm@C_{88}

To study the structural deformation of the Sm@C_{88} molecules we spaced them from each other to form a periodic crystal structure, and used bis(ethylenedithio)tetrthiafulvalene (ET) to hinder the rotation of the carbon cage[S1,S2]. ET has been used to cocrytallize with the EMF Sm@C_{80} for examing the structure of Sm@C_{80}[S3]. We mixed the isolated Sm@C_{88} and ET in toluene solution to form crystals of Sm@C_{88}·ET·0.5(toluene), which were used for the structural characterization at ambient pressure. The Sm@C_{88} carbon cage has C_s symmetry. We expect that the crystal should be similar to Sm@C_{80}·ET·0.5(toluene) in structure because of the close cage size. We thus constructed a crystal model based on the atomic coordination of Sm@C_{80}·ET·0.5(toluene)[S3] by replacing Sm@C_{80} with Sm@C_{88}. The crystal model was then refined by using the Rietveld method with anisotropic thermal parameters for non-hydrogen atoms and isotropic thermal parameters for hydrogen atoms. The refined XRD and crystal structure of Sm@C_{88}·ET·0.5(toluene) are shown in Fig. S1. The crystal is crystallized in a monoclinic structure with space group $P2_1/c$, and cell parameters of a =12.59(8) Å, b =22.74(2) Å, c=23.43(3) Å, β=107.18(2) °， V=6413.79(6) $Å^3$, Z = 4; D_c =1.61(6) gcm^{-3}. The values R_{wp}=5.97%, R_p=9.27% were achieved, suggesting that the constructed structure of Sm@C_{88}·ET·0.5(toluene)is reasonable. The shortest distance S(ET)···C(Sm@C_{88}) (3.13 Å) is shorter than the sum of the van der Waals radii of the S and C atoms (3.57 Å), which indicates a possible weak charge transfer between Sm@C_{88} and the ET molecules. The charge-transfer interaction may be weak because of the relatively weak electron donor ability of ET[S1,S2]. The closest C···C distance between the Sm@C_{88} and toluene molecules is 3.51 Å, which is close to the sum of the van der Waals radii of two C atoms, 3.40 Å.

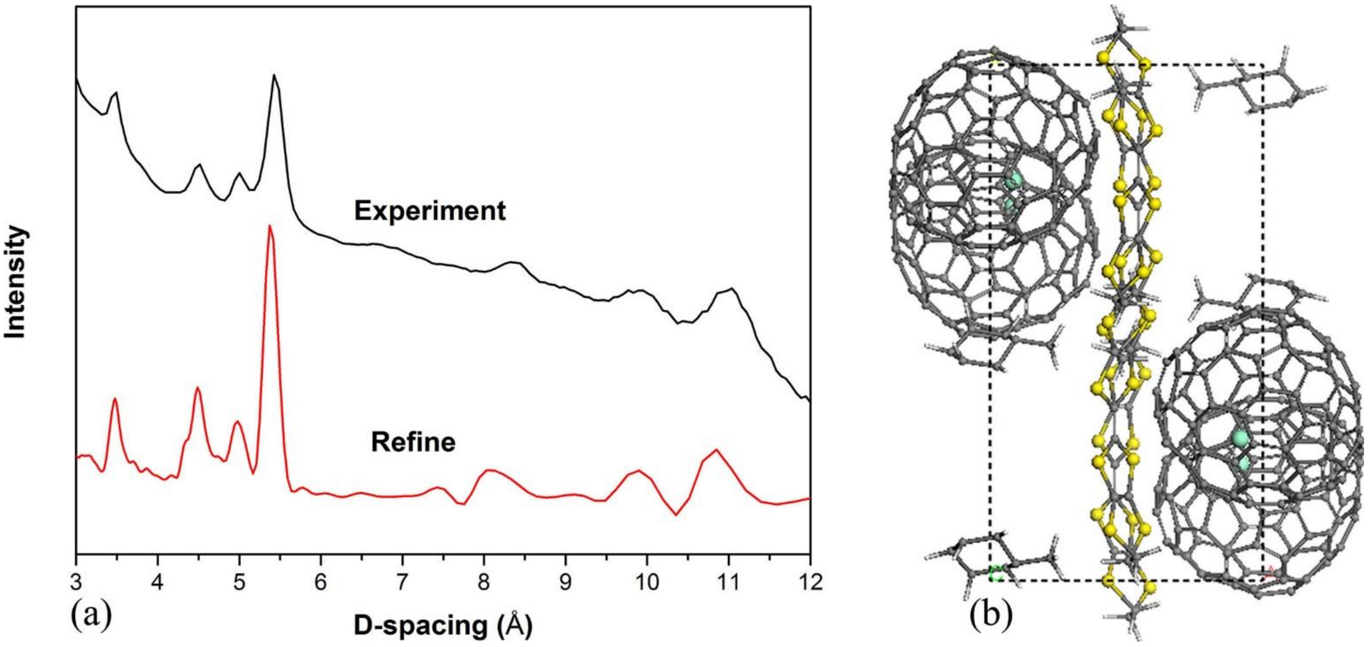

Figure S1 | (a) XRD pattern of Sm@C_{88}·ET·0.5(toluene) from experiment (black) and refine (red). (b) Crystalline Sm@C_{88}·ET·0.5(toluene)

Part 2. HRTEM study of Sm@C_{88}

To further study the microstructures of decompressed sample, we measured the HRTEM images of the samples at ambient pressure and decompressed from 18 GPa, shown in Fig. S2. The figures show that the sample at ambient pressure is an order phase, and the decompressed sample is amorphized. This indicates that the sample transforms to amorphization at 18 GPa and can be quenched to ambient pressure, in agreement with our IR results.

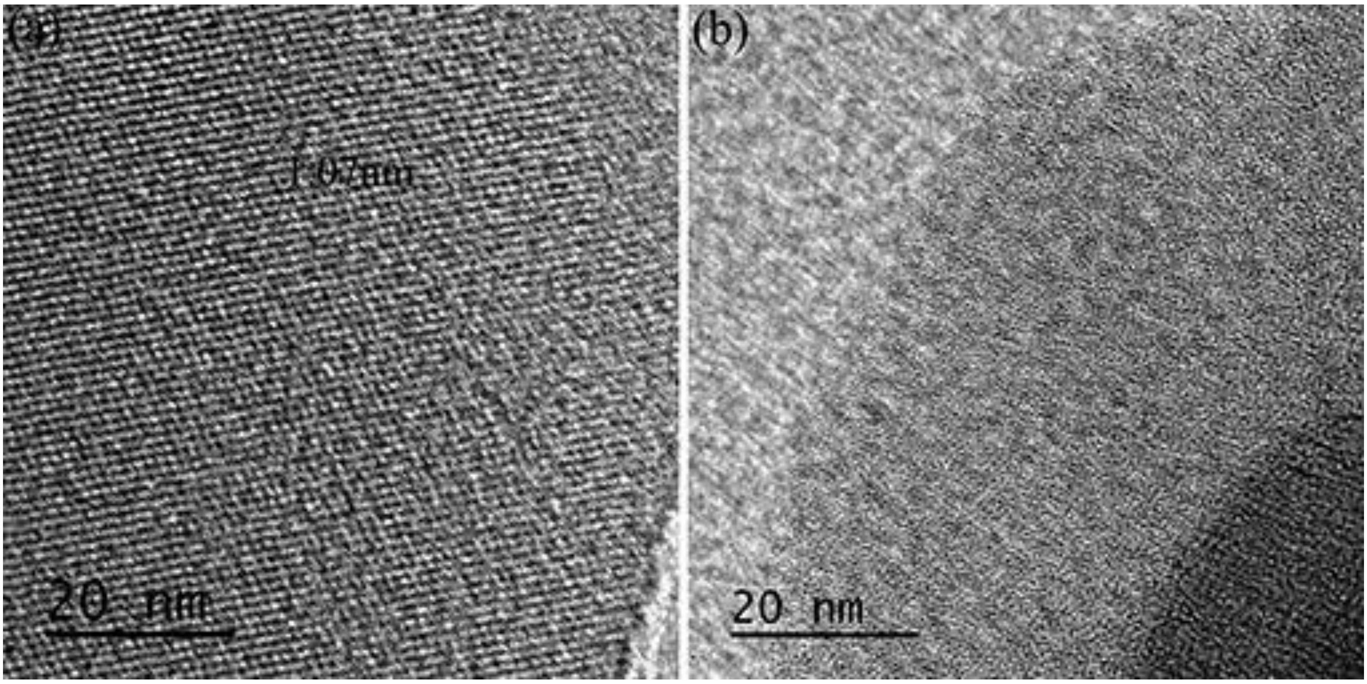

Figure S2 | HRTEM images of (a) the samples at ambient pressure and (b) decompressed from 18GPa.

Part 3. References for Supplementary Information

S1. Spitsina, N. G., Motyakin, M. V., Bashkin, I. V., Meletov, K. P. C_{60} fullerene and its molecular complexes under axial and shear deformation. *J. Phys.: Condens. Matter* **14,** 11089-11092 (2002).

S2. Izuoka, A. et al. An X-Ray crystallographic analysis of a $(BEDT\text{-}TTF)_2C_{60}$ charge-transfer complex. *J. Chem. Soc., Chem. Commun.* 1472-1473 (1992).

S3. Yang, H. et al. Isolation and crystallographic characterization of Sm@C_{2v}(3)-C_{80} through cocrystal formation with Ni^{II}(octaethylporphyrin) or bis(ethylenedithio)tetrathiafulvalene. *Inorg. Chem.* **52,** 1275-1284 (2013).
